# Relationship between cognitive function and prevalence of decrease in intrinsic academic motivation in adolescents

**DOI:** 10.1186/1744-9081-7-4

**Published:** 2011-01-14

**Authors:** Kei Mizuno, Masaaki Tanaka, Sanae Fukuda, Kyoko Imai-Matsumura, Yasuyoshi Watanabe

**Affiliations:** 1 Department of Physiology, Osaka City University Graduate School of Medicine, 1-4-3 Asahimachi, Abeno-ku, Osaka 545-8585, Japan; 2 Molecular Probe Dynamics Laboratory, RIKEN Center for Molecular Imaging Science, 6-7-3 Minatojima-minamimachi, Chuo-ku, Kobe City, Hyogo 650-0047, Japan; 3 Department of Medical Science on Fatigue, Osaka City University Graduate School of Medicine, 1-4-3 Asahimachi, Abeno-ku, Osaka 545-8585, Japan; 4 Department of Clinical, Health and Special Needs Education Needs, Hyogo University of Teacher Education Graduate School of Education, 942-1 Shimokume, Kato City, Hyogo 673-1494, Japan

## Abstract

**Background:**

Decrease in intrinsic motivation is a common complaint among elementary and junior high school students, and is related to poor academic performance. Since grade-dependent development of cognitive functions also influences academic performance by these students, we examined whether cognitive functions are related to the prevalence of decrease in intrinsic academic motivation.

**Methods:**

The study group consisted of 134 elementary school students from 4th to 6th grades and 133 junior high school students from 7th to 9th grades. Participants completed a questionnaire on intrinsic academic motivation. They also performed paper-and-pencil and computerized cognitive tests to measure abilities in motor processing, spatial construction, semantic fluency, immediate memory, short-term memory, delayed memory, spatial working memory, and selective, alternative, and divided attention.

**Results:**

In multivariate logistic regression analyses adjusted for grade and gender, scores of none of the cognitive tests were correlated with the prevalence of decrease in intrinsic academic motivation in elementary school students. However, low digit span forward test score and score for comprehension of the story in the *kana *pick-out test were positively correlated with the prevalence of decrease in intrinsic academic motivation in junior high school students.

**Conclusions:**

The present findings suggest that decrease in capacity for verbal memory is associated with the prevalence of decrease in intrinsic academic motivation among junior high school students.

## Background

Motivation, one of the most important psychological concepts in education, can be classified into intrinsic and extrinsic forms; intrinsic motivation refers to doing something because it is inherently interesting or enjoyable, while extrinsic motivation refers to doing something because it leads to a separable outcome [1]. It has been shown that intrinsic academic motivation results in better educational outcomes, such as higher academic performances, better quality of learning, increased persistence and effort in studies, and better psychological adjustment of learners, than does extrinsic motivation [2,3].

When students proceed to junior high school from elementary school, rapid changes in the environment occur, which may cause various behavioral and emotional problems [4]. One example is the number of Japanese students with school refusal, which was observed in 7,483 of 1,192,343 6th-graders and 22,566 of 1,199,764 7th-graders in 2005 [5]. It has been reported that the intrinsic academic motivation of school students also markedly decreases from elementary school to junior high school [6]. Identifying intrinsic academic motivation-related factors is thus important for preventing decrease in intrinsic academic motivation during this transition period.

Intrinsic academic motivation must engage the working memory system to relate achievements to an ultimate goal. This is especially true during learning, which serves to maintain "on tap" a limited amount of currently relevant information so that it is available for immediate use [7-9]. Function of working memory is included in executive function, which is defined as the set of mental control cognitive processes that permit goal-directed behaviour and develops dramatically between childhood and adolescence [10,11]. In studies on the development of executive function, for example, age-related gain has been reported in working memory [12,13], inhibitory control [14], task switching [15], adaptive problem solving [16], and various other planning and problem-solving tasks [17].

The development of executive function is observed in association with structural changes in the brain. Morphological analyses in children and adolescents have shown that brain maturation occurs at different rates in different brain regions: the primary sensory and motor areas are the first to complete development, while the association areas, especially in the frontal and parietal regions, are the last to mature [18,19]. Maturation of the frontal and parietal regions is of great importance for adequate processing of executive function. Executive function is also related to control of attention [20], an important element of information processing that is embodied in the central executive component in theoretical conceptions of working memory [21]. Attentional competency develops steadily through early and late childhood, perhaps due in part to the development of core processing resources [22,23]. In the literature on normal cognitive development, there are two general hypotheses: that as children grow older, they have more resources [24], or that they are able to utilize their existing resources more efficiently [25]. With more resources or increasing control over resources, children become able to pay attention to more stimuli, allocate their attention more efficiently in accordance with task demands, and generally use and benefit from more sophisticated strategies in complex activities such as dual tasks [26].

Recently, we reported the differences in development of several cognitive functions between elementary school and junior high school students using paper-and-pencil and computerized cognitive function tests which are used in the present study as well [10]. Although the performances of school students in a selective attention task [task C on the modified advanced trail making test (mATMT)] and a motor processing task (task D on the mATMT) improved by grade in elementary school, they did not change from elementary school to junior high school. In contrast, task performances of school students in short-term memory (digit span forward test), spatial working memory tasks (symbol digit modalities test and tasks A and B on the mATMT), semantic fluency test, alternative attention task (task E on the mATMT), and divided attention task (*kana *pick-out test) improved from elementary to junior high school. In junior high school, task performances of school students in semantic fluency and *kana *pick-tout tests improved by grade. These findings indicate that semantic fluency and divided attention develop from childhood to late adolescence. Based on these findings, we performed the present study; these cognitive tests were advantageous in terms of evaluation of cognitive development in children and adolescents.

A number of studies have shown relationships between motivation and cognitive functions [27-29]. However, these studies focused only on motivation induced by extrinsic reward (e.g., monetary reward). Only a few studies have attempted to clarify the relationships between intrinsic motivation and cognitive functions, and found that intrinsic motivation is associated with abilities in problem solving [30] and verbal memory consolidation [31]. However, in the transition period from elementary school to junior high school, development of executive functions [11], marked decrease in intrinsic academic motivation [6], and various behavioral and emotional problems in learning [4] do occur. In this transition period, several cognitive functions such as short-term and spatial working memories, semantic fluency, and alternative and divided attention develop [10]. We therefore hypothesized that intrinsic academic motivation is associated with grade-dependent development of cognitive functions in elementary school and junior high school students. However, the relationships between decrease in intrinsic academic motivation and cognitive functions have not been identified, i.e., cross-sectional studies of children and adolescents to examine these relationships have not been performed. We therefore administered cognitive function tests to 4th-, 5th-, and 6th-grade students in an elementary school and 7th-, 8th-, and 9th-grade students in a junior high school and investigated the relationships between cognitive functions and intrinsic academic motivation.

## Methods

### Participants

Participants were recruited from 4th, 5th, and 6th grades of an elementary school and 7th, 8th, and 9th grades of a junior high school in Hyogo Prefecture, Japan. Most of the students in the elementary school proceed on to the junior high school. Forty-five participants with medical illnesses, such as allergic disease, bronchial asthma, thyroid disease, nephritis, diabetes mellitus, heart disease, anemia, myodystrophy, and epilepsy, were excluded from the analyses. In addition, 50 participants who did not complete the questionnaire on intrinsic academic motivation were excluded. Motivation score on the questionnaire and performance on the cognitive function tests in a total of 267 elementary school and junior high school students were analyzed in the present study, including 45 4th- [23 females, 9.8 ± 0.4 years of age (mean ± SD)], 46 5th- (24 females; 10.7 ± 0.5 years of age) and 43 6th-grade students (22 females; 11.7 ± 0.5 years of age) in the elementary school, and 51 7th- (32 females; 12.6 ± 0.5 years of age), 32 8th- (8 females; 13.4 ± 0.5 years of age), and 50 9th-grade students (23 females; 14.6 ± 0.5 years of age) in the junior high school. The protocol was approved by the Ethics Committee of Osaka City University. All participants and their parents gave written informed consent for participation in the study.

### Intrinsic academic motivation scale

The extent of intrinsic academic motivation was measured using the intrinsic motivation scale toward learning [32]. The reliability and validity of the Japanese scale for evaluating the level of intrinsic academic motivation in school students were previously confirmed [33]. The intrinsic academic motivation scale consisted of 30 questions using a 4-level (1-4) scale and was constructed as a continuous variable. Example items from the intrinsic academic motivation scale are "Does the child like hard challenging work?", "Does the child work to satisfy his or her own interest and curiosity?", and "Does the child prefer to work and figure out problems on his/her own?". The total score for the 30-item intrinsic academic motivation scale ranges from 30 to 120, with higher scores indicating a greater degree of intrinsic academic motivation. Based on a previous epidemiological report, lowering of academic motivation was defined as a score equal to or lower than 77 on the Japanese version of the intrinsic motivation scale toward learning [6].

### Cognitive function tests

Participants performed paper-and-pencil and computerized cognitive tests [10]. The paper-and-pencil cognitive tests consisted of a list learning test [34], *kana *pick-out test [35], semantic fluency test [36], figure copying test [34], digit span forward test [37], symbol digit modalities test [38], and list recall test [34]. The paper-and-pencil cognitive tests were performed in this order.

The list learning test was used to assess immediate memory. This test consists of immediate recall of a 10-item list of words over four learning trials. The words are semantically unrelated, characterized by early age of acquisition, relatively high-imagery, and as phonetically unique as possible. The full score for this test is 40.

The *kana *pick-out test requires parallel processing of reading and picking out of letters, and also requires appropriate allocation of attentional resources to the two activities. Participants are shown a short story written in Japanese *kana *characters. They are required to find as many vowel symbols as possible within 2 min, while understanding the meaning of the story. Two min after the start of the test, they are asked 10 questions about the contents of the story over a 2 min period. The Japanese *kana *characters consist of 66 phonetic symbols that include five vowels; the story consists of 406 symbols with 61 vowels. *Kana *pick-out score is calculated as count for *kana *pick-out number subtracted by *kana *omission number. The full *kana *pick-out score for this test is 61, with a full comprehension score of 10.

The semantic fluency test was used to assess retrieval of learned material. This test consists of the total number of exemplars generated for a given semantic category (vegetables) within 1 min. The semantic categories were chosen in an attempt to minimize retrieval demands and thereby more specifically tap semantic stores rather than retrieval strategies.

The figure copying test was used to assess spatial construction ability. This test consists of copying of a geometric figure comprised of 10 parts. Each part receives a 2-point score (accuracy and placement), for a total of 20 possible points, and thus a possible full score of 20. The time limit for performance of the test is set at 4 min.

The digit span forward test was used to assess short-term memory. An experimenter read a series of digits at the rate of approximately one number per second and asked the participants to write on paper the digits exactly as read. The series ranged from 4 to 8 digits in length and were presented in order of increasing numbers of digits. The full score for this test is 8.

The cognitive demand of the symbol digit modalities test includes spatial working memory and visual scanning. The test consists of 1) a key with two rows, with nine stimulus symbols in the upper row, and matched numbers (1 - 9) in the lower row, and 2) a two-row grid with the same nine stimulus symbols in the upper row and 84 blank cells for numeric responses in the lower row. The full score for this test is 84. The time limit for performance of this test is set at 1 min and 30 s.

The list recall test was used to assess delayed memory. This test involves free recall of the words from the list learning test. The list recall test was performed 30 min after the list learning test. The full score for this test is 10.

The computerized cognitive test consisted of mATMT and separate tasks A, B, C, D, and E [39,40]. For the mATMT, participants performed visual search trials. In this test, circles numbered from 1 to 25 are randomly located on the screen of a personal computer, and participants are required to use a computer mouse to click on these circles in sequence, starting with circle number 1. In task A, when the participant clicks on a target circle, the positions of the other circles also remain the same. In task B, when the participant clicks on the first target circle, a new circle number 26 appears on the screen. The positions of the other circles remain the same. Thus, tasks A and B require spatial working memory. The procedure for task C is the same as that for task B, except that the positions of the other circles randomly change after each target circle is clicked on. Thus, task C requires selective attention alone. In task D, circles numbered from 1 to 25 are regularly and sequentially located on a computer screen. Thus, task D requires no visual search, and only motor processing. In task E, circles numbered from 1 to 13 and 12 *kana *letters (Japanese phonograms) are randomly located on the screen, and participants are required to use a computer mouse to alternately click on numbers and *kana*; this task thus requires alternative attention. Participants performed tasks A, B, C, D, and E in this order, and time limits for performance of the mATMT were set at 10 min. Participants were instructed to perform the tasks as quickly and correctly as possible.

### Experimental design

The questionnaire on intrinsic motivation toward learning was distributed to the participants in a classroom at their school before the cognitive tests. An explanation was given to participants of the rules for each paper-and-pencil cognitive test in the classroom. Before the participants performed each cognitive test, they practiced the test for a period of 1 min. One half of the participants performed the paper-and-pencil cognitive tests in the classroom for around 35 min. After the participants completed the tests, they moved to a computer room and performed the mATMT within 10 min. The remaining participants first performed the mATMT in the computer room and then moved to the classroom and performed the paper-and-pencil cognitive tests.

### Statistical analyses

Comparisons of intrinsic academic motivation scores among the six grade groups were performed using one-way analysis of variance (ANOVA). When statistically significant effects were found, intergroup differences among the 6 grade groups were evaluated using the two-tailed Student's t-test with Bonferroni correction. Relationships between cognitive function score and grade and the relationships between cognitive function and intrinsic academic motivation scores were estimated using Spearman's rank correlation test and Pearson's correlation test, respectively. Categorical variables were compared using the χ^2 ^test. Differences in mean values between the participants who were not motivated and those who were motivated were compared using the two-tailed Student's t-test. Univariate and multivariate logistic regression analyses were performed to identify factors associated with the prevalence of decrease in intrinsic academic motivation. We calculated the 95% confidence interval (CI) for each odds ratio (OR). In the analyses, the number of cases varied due to incidental missing values. *p *values less than .05 were considered statistically significant. Statistical analyses were performed using the SPSS 17.0 software package (SPSS, Chicago, IL).

## Results

Intrinsic academic motivation scores of the six grades are shown in Table 1. One-way ANOVA revealed a main effect of grade on intrinsic academic motivation score (*F *= 3.70, *p *= .003). Although the intrinsic academic motivation scores among the 4th-, 5th-, and 6th-grade students in the elementary school were similar, the intrinsic academic motivation scores of the 7th- and 8th-grade students in the junior high school were significantly lower than those of the 4th-grade students in the elementary school. The intrinsic academic motivation score of the 9-th grade students in the junior high school also tended to be lower than that of the 4th-grade students in the elementary school. Intrinsic academic motivation scores did not differ among the 7th-, 8th-, and 9th-grade students in the junior high school.

**Table 1:** Intrinsic academic motivation scores in the six grades

	Elementary school			Junior high school		
		
	4th grade	5th grade	6th grade	7th grade	8th grade	9th grade
Intrinsic academic motivation score	90.4 ± 14.7	89.6 ± 13.4	88.4 ± 14.1	82.4 ± 0.5*	81.2 ± 12.9*	83.5 ± 11.7^#^

Values are presented as the mean ± SD. * *p *< .05, significantly different from the corresponding values for the 4th-grade group; ^#^*p *< .1, different from the corresponding values for the 4th-grade group (one-way analysis of variance, followed by Student's t-test with Bonferroni correction).

Results of the correlation analyses between cognitive function scores and grade or intrinsic academic motivation scores are shown in Table 2. Scores of all of the paper-and-pencil cognitive tests were positively correlated with grade in the elementary school and junior high school students. In the mATMT, reaction times on all the tasks were negatively correlated with the grade of students. Although the scores for symbol digit modalities and semantic fluency tests were positively correlated with the intrinsic academic motivation score in elementary school students, the scores of other paper-and-pencil cognitive tests were not correlated with the intrinsic academic motivation score in these students. Negative correlations between the reaction times on all the tasks of the mATMT and intrinsic academic motivation score were observed in the elementary school students. In the junior high school students, the scores for list learning and list recall tests, digit span forward test, and comprehension of the story in the *kana *pick-out test were positively correlated with intrinsic academic motivation score. No significant correlations between task performances on the other cognitive tests or intrinsic academic motivation score were observed in the junior high school students.

**Table 2:** Correlations between cognitive function score and grade or intrinsic academic motivation score

		Intrinsic academic motivation score	
		
Cognitive function score	Grade [4th - 9th (n = 267)]	Elementary school [4th - 6th (n = 134)]	Junior high school [7th - 9th (n = 133)]
List learning test, score	.240**	.107	.209^†^
List recall test, score	253**	.086	.186^†^
Digit span forward test, score	.370**	.159	.262^††^
Symbol digit modalities test, score	.544**	.255^††^	.098
Semantic fluency test, score	.433**	.197^†^	.152
Figure copying test, score	.126*	.070	.152
*Kana *pick-out test			
*Kana *pick-out number	.555**	.117	.036
*Kana *omission number	-.044	-.014	-.040
*Kana *pick-out score	.529**	.102	.051
Score for story comprehension	.264**	.074	.264^††^
Modified advanced trail making test			
RT on task A, s	-.525**	-.102	-.112
RT on task B, s	-.501**	-.224^†^	-.095
RT on task C, s	-.476**	-.225^†^	-.107
RT on task D, s	-.419**	-.269^††^	-.135
RT on task E, s	-.461**	-.235^††^	-.144

RT, reaction time. Values are presented as Spearman's rank or Pearson's correlation coefficients. * *p *< .05, **p < .01, significant correlation (Spearman's rank correlation test); ^†^*p *< .05, ^††^*p *< .01, significant correlation (Pearson's correlation test).

Baseline characteristics of the study participants are summarized in Table 3 according to intrinsic academic motivation. The prevalences of decrease in intrinsic academic motivation among the elementary and junior high school students were 18.7% and 37.6%, respectively, indicating that the prevalence of decrease in intrinsic academic motivation in the junior high school students was approximately twice that in the elementary school students (χ^2 ^= 11.9, *p *< .001). Neither age nor gender differed between participants who were motivated and those who were not motivated in both the elementary and the junior high school. On cognitive function testing, reaction times on task C of the mATMT were slower for participants who were not motivated than for those who were motivated in the elementary school. In the elementary school, task performances on other cognitive tests in participants who were motivated and those who were not were similar. In the junior high school, digit span forward test score and score for story comprehension of the *kana *pick-out test were lower for participants who were not motivated than for those who were motivated. In the junior high school, task performances on other cognitive tests in participants who were motivated and those who were not did not differ.

**Table 3:** Baseline characteristics of study participants according to intrinsic academic motivation level

	Elementary school			Junior high school		
		
	Overall	Motivated	Not motivated	Overall	Motivated	Not motivated
	(n = 134)	(n = 109)	(n = 25)	(n = 133)	(n = 83)	(n = 50)
Age, years	10.8 ± 0.9	10.8 ± 0.9	10.8 ± 0.8	13.5 ± 1.0	13.6 ± 1.0	13.4 ± 0.9
Female (%)	69 (51.5)	55 (50.5)	14 (56.0)	63 (47.4)	38 (45.8)	25 (50.0)
List learning test, score	31.6 ± 3.8	31.8 ± 3.7	31.0 ± 4.5	33.0 ± 3.4	33.4 ± 3.3	32.3 ± 3.4
List recall test, score	8.4 ± 1.5	8.5 ± 1.5	8.3 ± 1.5	9.0 ± 1.2	9.1 ± 1.0	8.8 ± 1.4
Digit span forward test, score	5.7 ± 1.0	5.7 ± 1.0	5.6 ± 1.0	6.4 ± 1.0	6.6 ± 1.0	6.0 ± 1.0***
Symbol digit modalities test, score	44.6 ± 11.8	45.3 ± 11.8	41.7 ± 11.6	57.6 ± 10.5	59.0 ± 9.9	55.2 ± 11.2
Semantic fluency test, score	9.7 ± 2.3	9.8 ± 2.2	9.1 ± 2.7	11.4 ± 3.1	11.8 ± 3.1	10.9 ± 3.0
Figure copying test, score	15.5 ± 5.9	15.4 ± 6.2	15.8 ± 4.3	14.7 ± 7.5	15.5 ± 7.0	13.4 ± 8.3
*Kana *pick-out test						
*Kana *pick-out number	27.1 ± 8.8	27.4 ± 9.0	25.7 ± 7.7	38.0 ± 9.3	38.5 ± 9.3	37.3 ± 9.4
*Kana *omission number	7.3 ± 6.7	7.4 ± 7.0	7.0 ± 5.1	6.8 ± 6.0	6.7 ± 6.6	6.9 ± 4.8
*Kana *pick-out score	19.7 ± 10.9	20.0 ± 11.5	18.7 ± 7.9	31.3 ± 11.3	31.8 ± 12.2	30.4 ± 9.8
Score for story comprehension	3.6 ± 1.8	3.7 ± 1.8	3.4 ± 1.7	4.7 ± 2.1	5.2 ± 2.1	4.0 ± 2.1**
Modified advanced trail making test						
RT on task A, s	44.5 ± 12.6	44.4 ± 12.6	45.2 ± 12.7	35.3 ± 7.8	34.5 ± 7.7	36.5 ± 7.9
RT on task B, s	67.6 ± 19.9	67.1 ± 19.2	69.4 ± 22.8	51.5 ± 14.5	50.6 ± 13.4	53.1 ± 16.2
RT on task C, s	91.5 ± 26.2	89.2 ± 22.9	101.4 ± 36.0*	72.5 ± 17.1	70.6 ± 15.0	75.8 ± 19.7
RT on task D, s	14.9 ± 3.9	14.6 ± 3.7	16.0 ± 4.6	12.3 ± 3.5	12.1 ± 3.9	12.7 ± 2.7
RT on task E, s	76.4 ± 30.4	74.0 ± 24.9	86.8 ± 46.7	54.6 ± 15.6	52.8 ± 15.3	57.6 ± 15.8

RT, reaction time. Values are presented as the mean ± SD or number (%). *p < .05, ** *p *< .01, *** *p *< .001, significantly different from the corresponding values for the motivated group.

Univariate logistic regression analyses were performed to identify cognitive functions associated with the prevalence of decreased intrinsic academic motivation in elementary school students (Table 4). None of grade, gender, and scores or reaction times for cognitive tests was correlated with the prevalence of decreased intrinsic academic motivation. Multivariate logistic regression analyses adjusted for grade and gender also revealed that scores or reaction times of none of the cognitive tests were correlated with the prevalence of decreased intrinsic academic motivation (Table 4).

**Table 4:** Univariate and multivariate logistic regression analyses of the prevalence of decrease in intrinsic academic motivation in elementary school students

	OR (95% CI)	
	
	Crude	Multiple-Adjusted*
Grade		
4th grade	Reference (1.00)	
5th grade	0.60 (0.19 - 1.85)	
6th grade	1.21 (0.44 - 3.35)	
*p *for trend	0.455	
Female gender	0.80 (0.33 - 1.92)	
List learning test		
Tertile 1 (≥34)	Reference (1.00)	Reference (1.00)
Tertile 2 (30 - 33)	1.86 (0.66 - 5.26)	1.71 (0.59 - 4.91)
Tertile 3 (≤30)	0.91 (0.26 - 3.14)	0.88 (0.25 - 3.08)
*p *for trend	0.347	0.441
List recall test		
Tertile 1 (≥10)	Reference (1.00)	Reference (1.00)
Tertile 2 (8 - 9)	0.99 (0.33 - 2.95)	0.93 (0.31 - 2.83)
Tertile 3 (≤7)	1.56 (0.46 - 5.31)	1.42 (0.41 - 4.99)
*p *for trend	0.679	0.748
Digit span forward test		
Tertile 1 (≥7)	Reference (1.00)	Reference (1.00)
Tertile 2 (6)	2.02 (0.46 - 8.90)	2.16 (0.47 - 9.91)
Tertile 3 (≤5)	1.14 (0.28 - 4.67)	1.16 (0.28 - 4.83)
*p *for trend	0.521	0.483
Symbol digit modalities test		
Tertile 1 (≥48)	Reference (1.00)	Reference (1.00)
Tertile 2 (39 - 47)	1.39 (0.44 - 4.42)	1.55 (0.48 - 5.04)
Tertile 3 (≤38)	1.47 (0.50 - 4.38)	1.68 (0.53 - 5.29)
*p *for trend	0.762	0.646
Semantic fluency test		
Tertile 1 (≥11)	Reference (1.00)	Reference (1.00)
Tertile 2 (9 - 10)	1.09 (0.33 - 3.56)	1.20 (0.36 - 4.03)
Tertile 3 (≤8)	2.28 (0.79 - 6.56)	2.49 (0.84 - 7.34)
*p *for trend	0.239	0.210
Figure copying test		
Tertile 1 (≥19)	Reference (1.00)	Reference (1.00)
Tertile 2 (16 - 18)	0.77 (0.25 - 2.34)	0.79 (0.25 - 2.48)
Tertile 3 (≤15)	1.48 (0.48 - 4.50)	1.66 (0.53 - 5.23)
*p *for trend	0.484	0.420
*Kana *pick-out test		
*Kana *pick-out number		
Tertile 1 (≥30)	Reference (1.00)	Reference (1.00)
Tertile 2 (24 - 29)	1.48 (0.47 - 4.68)	1.69 (0.52 - 5.49)
Tertile 3 (≤23)	2.22 (0.71 - 6.97)	2.80 (0.83 - 9.43)
*p *for trend	0.389	0.250
*Kana *omission number		
Tertile 1 (≤3)	Reference (1.00)	Reference (1.00)
Tertile 2 (4 - 7)	1.07 (0.35 - 3.31)	1.10 (0.35 - 3.44)
Tertile 3 (≥8)	0.91 (0.30 - 2.78)	0.84 (0.27 - 2.63)
*p *for trend	0.955	0.889
*Kana *pick-out score		
Tertile 1 (≥24)	Reference (1.00)	Reference (1.00)
Tertile 2 (16 - 23)	1.06 (0.34 - 3.34)	1.14 (0.35 - 3.68)
Tertile 3 (≤15)	1.40 (0.47 - 4.19)	1.55 (0.50 - 4.78)
*p *for trend	0.806	0.730
Score for comprehension of the story		
Tertile 1 (≥5)	Reference (1.00)	Reference (1.00)
Tertile 2 (3 - 4)	1.38 (0.45 - 4.28)	1.37 (0.44 - 4.29)
Tertile 3 (≤2)	1.55 (0.48 - 4.99)	1.54 (0.47 - 5.05)
*p *for trend	0.749	0.763
Modified advanced trail making test		
RT on task A		
Tertile 1 (<39.1)	Reference (1.00)	Reference (1.00)
Tertile 2 (39.1 - 47.8)	2.35 (0.83 - 6.63)	2.18 (0.75 - 6.34)
Tertile 3 (≥47.9)	0.62 (0.17 - 2.30)	0.59 (0.15 - 2.35)
*p *for trend	0.064	0.085
RT on task B		
Tertile 1 (<57.9)	Reference (1.00)	Reference (1.00)
Tertile 2 (57.9 - 73.2)	0.60 (0.20 - 1.84)	0.69 (0.22 - 2.18)
Tertile 3 (≥73.3)	0.78 (0.27 - 2.20)	0.89 (0.28 - 2.81)
*p *for trend	0.667	0.813
RT on task C		
Tertile 1 (<80.2)	Reference (1.00)	Reference (1.00)
Tertile 2 (80.2 - 98.2)	0.79 (0.24 - 2.58)	0.85 (0.25 - 2.89)
Tertile 3 (≥98.3)	1.67 (0.58 - 4.84)	1.83 (0.58 - 5.72)
*p *for trend	0.371	0.358
RT on task D		
Tertile 1 (<12.7)	Reference (1.00)	Reference (1.00)
Tertile 2 (12.7 - 16.4)	1.39 (0.46 - 4.25)	1.74 (0.52 - 5.75)
Tertile 3 (≥16.5)	1.57 (0.53 - 4.67)	2.14 (0.61 - 7.51)
*p *for trend	0.711	0.481
RT on task E		
Tertile 1 (<65.5)	Reference (1.00)	Reference (1.00)
Tertile 2 (64.5 - 79.7)	1.23 (0.42 - 3.55)	1.31 (0.44 - 3.85)
Tertile 3 (≥79.8)	0.93 (0.30 - 2.83)	0.89 (0.28 - 2.81)
*p *for trend	0.870	0.794

* Adjusted for grade and gender. RT, reaction time; OR, odds ratio; CI, confidence interval.

Likewise, univariate logistic regression analyses were performed for the junior high school students (Table 5). Neither grade nor gender was correlated with the prevalence of decrease in intrinsic academic motivation. List learning score ([Tertile 2 (32-34) vs. Tertile 1 (≥35); OR: 2.90, 95% CI: 1.20 to 6.97; Tertile 3 (≤31) vs. Tertile 1 (≥35); OR: 2.45, 95% CI: 0.95 to 6.35; *p *= .029]), digit span forward test score ([Tertile 2 (6) vs. Tertile 1 (≥7); OR: 3.41, 95% CI: 1.42 to 8.15; Tertile 3 (≤5) vs. Tertile 1 (≥7); OR: 5.55, 95% CI: 2.10 to 14.69; *p *= .001]), and score for comprehension of the story in the *kana *pick-out test ([Tertile 2 (4-5) vs. Tertile 1 (≥6); OR: 2.81, 95% CI: 1.14 to 6.69; Tertile 3 (≤3) vs. Tertile 1 (≥6); OR: 4.47, 95% CI: 1.73 to 11.56; *p *= .007]) were each positively correlated with the prevalence of decrease in intrinsic academic motivation. Scores or reaction times of none of the other cognitive tests were correlated with the prevalence of decrease in intrinsic academic motivation. Multivariate logistic regression analyses adjusted for grade and gender were also performed (Table 5). Digit span forward test score ([Tertile 2 (6) vs. Tertile 1 (≥7); OR: 3.41, 95% CI: 1.39 to 8.33; Tertile 3 (≤5) vs. Tertile 1 (≥7); OR: 5.10, 95% CI: 1.88 to 13.86; *p *= .003]) and score for comprehension of the story in the *kana *pick-out test ([Tertile 2 (4-5) vs. Tertile 1 (≥6); OR: 2.95, 95% CI: 1.17 to 7.46; Tertile 3 (≤3) vs. Tertile 1 (≥6); OR: 4.95, 95% CI: 1.82 to 13.43; *p *= .006]) were positively correlated with the prevalence of decrease in intrinsic academic motivation in the junior high school students. Scores or reaction times of none of the other cognitive tests were associated with the prevalence of decrease in intrinsic academic motivation.

**Table 5:** Univariate and multivariate logistic regression analyses of the prevalence of decrease in intrinsic academic motivation in junior high school students

	OR (95% CI)	
	
	Crude	Multiple-Adjusted*
Grade		
7th grade	Reference (1.00)	
8th grade	1.03 (0.42 - 2.50)	
9th grade	0.51 (0.22 - 1.18)	
*p *for trend	0.212	
Female gender	0.84 (0.42 - 1.71)	
List learning test		
Tertile 1 (≥35)	Reference (1.00)	Reference (1.00)
Tertile 2 (32 - 34)	2.90 (1.20 - 6.97)	2.77 (1.12 - 6.82)
Tertile 3 (≤31)	2.45 (0.95 - 6.35)	2.34 (0.89 - 6.13)
*p *for trend	0.048	0.072
List recall test		
Tertile 1 (≥10)	Reference (1.00)	Reference (1.00)
Tertile 2 (9)	0.56 (0.22 - 1.47)	0.55 (0.2 - 1.46)
Tertile 3 (≤8)	1.76 (0.77 - 4.05)	1.78 (0.76 - 4.17)
*p *for trend	0.086	0.078
Digit span forward test		
Tertile 1 (≥7)	Reference (1.00)	Reference (1.00)
Tertile 2 (6)	3.41 (1.42 - 8.15)	3.41 (1.39 - 8.33)
Tertile 3 (≤5)	5.55 (2.10 - 14.69)	5.10 (1.88 - 13.86)
*p *for trend	0.001	0.003
Symbol digit modalities test		
Tertile 1 (≥62)	Reference (1.00)	Reference (1.00)
Tertile 2 (54 - 61)	0.90 (0.35 - 2.32)	0.87 (0.33 - 2.25)
Tertile 3 (≤53)	2.11 (0.88 - 5.08)	2.04 (0.83 - 5.01)
*p *for trend	0.129	0.150
Semantic fluency test		
Tertile 1 (≥13)	Reference (1.00)	Reference (1.00)
Tertile 2 (10 - 12)	1.50 (0.59 - 3.78)	1.27 (0.49 - 3.31)
Tertile 3 (≤9)	2.01 (0.85 - 4.72)	1.80 (0.68 - 4.72)
*p *for trend	0.281	0.489
Figure copying test		
Tertile 1 (≥20)	Reference (1.00)	Reference (1.00)
Tertile 2 (17 - 19)	0.99 (0.41 - 2.38)	1.11 (0.44 - 2.77)
Tertile 3 (≤16)	1.57 (0.63 - 3.93)	2.01 (0.72 - 5.57)
*p *for trend	0.494	0.324
*Kana *pick-out test		
*Kana *pick-out number		
Tertile 1 (≥43)	Reference (1.00)	Reference (1.00)
Tertile 2 (34 - 42)	1.37 (0.58 - 3.26)	1.23 (0.50 - 2.98)
Tertile 3 (≤33)	1.22 (0.52 - 2.87)	0.95 (0.36 - 2.50)
*p *for trend	0.768	0.843
*Kana *omission number		
Tertile 1 (≤3)	Reference (1.00)	Reference (1.00)
Tertile 2 (4 - 7)	1.58 (0.65 - 3.85)	1.72 (0.70 - 4.27)
Tertile 3 (≥8)	1.91 (0.78 - 4.72)	1.86 (0.75 - 4.65)
*p *for trend	0.358	0.359
*Kana *pick-out score		
Tertile 1 (≥36)	Reference (1.00)	Reference (1.00)
Tertile 2 (28 - 35)	1.34 (0.56 - 3.22)	1.29 (0.52 - 3.16)
Tertile 3 (≤27)	1.83 (0.76 - 4.39)	1.67 (0.63 - 4.45)
*p *for trend	0.401	0.587
Score for comprehension of the story		
Tertile 1 (≥6)	Reference (1.00)	Reference (1.00)
Tertile 2 (4 - 5)	2.81 (1.14 - 6.96)	2.95 (1.17 - 7.46)
Tertile 3 (≤3)	4.47 (1.73 - 11.56)	4.95 (1.82 - 13.43)
*p *for trend	0.007	0.006
Modified advanced trail making test		
RT on task A		
Tertile 1 (<31.2)	Reference (1.00)	Reference (1.00)
Tertile 2 (31.2 - 36.2)	1.76 (0.71 - 4.38)	2.01 (0.77 - 5.25)
Tertile 3 (≥36.3)	2.52 (1.05 - 6.07)	2.44 (0.98 - 6.09)
*p *for trend	0.118	0.144
RT on task B		
Tertile 1 (<45.0)	Reference (1.00)	Reference (1.00)
Tertile 2 (45.0 - 54.7)	1.12 (0.48 - 2.62)	1.13 (0.47 - 2.74)
Tertile 3 (≥54.8)	1.39 (0.59 - 3.29)	1.23 (0.50 - 3.02)
*p *for trend	0.748	0.898
RT on task C		
Tertile 1 (<64.8)	Reference (1.00)	Reference (1.00)
Tertile 2 (64.8 - 78.9)	0.91 (0.39 - 2.15)	0.92 (0.38 - 2.23)
Tertile 3 (≥79.0)	1.56 (0.66 - 3.71)	1.42 (0.59 - 3.42)
*p *for trend	0.440	0.615
RT on task D		
Tertile 1 (<11.0)	Reference (1.00)	Reference (1.00)
Tertile 2 (11.0 - 12.8)	1.87 (0.76 - 4.62)	1.91 (0.76 - 4.79)
Tertile 3 (≥12.9)	2.41 (1.00 - 5.82)	2.52 (1.02 - 6.20)
*p *for trend	0.141	0.127
RT on task E		
Tertile 1 (<47.1)	Reference (1.00)	Reference (1.00)
Tertile 2 (47.1 - 57.7)	1.39 (0.58 - 3.31)	1.39 (0.56 - 3.40)
Tertile 3 (≥57.8)	1.97 (0.82 - 4.75)	1.89 (0.76 - 4.65)
*p *for trend	0.316	0.388

* Adjusted for grade and gender. RT, reaction time; OR, odds ratio; CI, confidence interval.

## Discussion

To our knowledge, the present research is the first cross-sectional study to determine the association between cognitive functions and the prevalence of decrease in intrinsic academic motivation in junior high school students. Our cross-sectional findings demonstrated that scores of none of the cognitive tests administered were associated with the prevalence of decrease in intrinsic academic motivation in elementary school students. In junior high school students, digit span forward test score and score for comprehension of the story in the *kana *pick-out test were associated with the prevalence of decrease in intrinsic academic motivation.

Previous research has suggested that discussions of performance outcomes can have important psychological consequences for children's motivation to achieve during the middle childhood years [41,30]. Mueller & Dweck [30] reported the relationship between intrinsic academic motivation and problem solving performance in late elementary school students. In the present study, although we did not measure problem solving ability, we did measure other executive functions. However, scores of none of the cognitive tests examined were associated with the prevalence of decrease in intrinsic academic motivation in late elementary school students in the present study. We therefore concluded that, other than problem solving, executive function was not specifically related to decrease in intrinsic academic motivation in late elementary school students.

In junior high school students, decrease in short-term memory or working memory capacity and comprehension level during divided attention were associated with the prevalence of decrease in intrinsic academic motivation. Swanson [42] noted that short-term memory is important in reading comprehension in children and adults. In the present study, we also found a positive correlation between the digit span forward test score and score for comprehension of the story in the *kana *pick-out test in junior high school students (*r *= .182, *p *= .038). These results suggest that capacity for verbal memory is related to the level of intrinsic academic motivation. In addition, scores for the list learning and list recall tests, digit span forward test, and comprehension of the story in the *kana *pick-out test were associated with the intrinsic academic motivation score in junior high school students. These findings also suggest that the capacity for verbal memory is associated with intrinsic academic motivation. Nielson & Bryant [31] reported that verbal memory consolidation was improved by intrinsic motivation in late adolescence. Intrinsic academic motivation may thus contribute not only to the reinforcement of short-term verbal memory but also to that of long-term verbal memory.

Recently, we found that task performances of the digit span forward test and *kana *pick-out test improved from elementary school to junior high school [10], suggesting that capacity for verbal memory and development of the frontal regions of the brain are related to the level of intrinsic academic motivation. The neural substrates activated in the digit span forward test have been identified in adults [43]. Activity in the dorsolateral prefrontal cortex during 7-digit trials was robust compared to that during 5-digit trials. In a previous study, although the mean number of digits in the digit span forward test in the 4th-, 5th-, and 6th-grade in elementary school students was around 5, that in the 9th-grade in junior high school students was around 7 [10]. These findings suggest that capacity for short-term memory may progress with the development of the dorsolateral prefrontal cortex from elementary to junior high school. The neural substrates of divided attention, which was tested using the *kana *pick-out test, were also identified using functional magnetic resonance imaging (fMRI) (unpublished observations). The *kana *pick-out test involves both linguistic skill and divided attention processing. We found that the left lateral prefrontal cortex plays a crucial role in the parallel processing required to perform the *kana *pick-out test. These findings clearly demonstrate that the dual task condition engages more attentional control than single task conditions due to greater and more complex demands on voluntary attentional resource allocation. Bunge et al. [44] reported measurement of neural activity using fMRI while participants performed two tasks: sentence reading and short-term memory for five words. Consistent with our results, performance of these dual tasks was associated with stronger activation in the left lateral prefrontal cortex than was performance of single tasks alone. These findings suggest that divided attention may progress with the development of the lateral prefrontal cortex from elementary to junior high school.

Previous neuroimaging studies demonstrated that the lateral prefrontal, anterior cingulate, and temporal cortices were associated with verbal memory [45,46]. In particular, increased activation in the anterior cingulate cortex was closely related to the capacity for verbal memory [46]. Therefore, attention controlling system, supported by the anterior cingulate cortex, may play primary roles to sustain capacity for verbal memory. Neuroimaging studies also demonstrated that motivation affects the enhancement of neural activities in brain regions related to performance of several cognitive functions [9,27,28,42]. Hence, one might expect that a study of intrinsic academic motivation will result in enhancement of activity of some brain regions such as anterior cingulate cortex in order to construct the behavior reinforcement for better academic performance such as learning in junior high school students.

Severe lowering of intrinsic academic motivation is associated with impairment of cognitive functions and academic performance. In fact, impairment of executive function is observed in depressive disorder [47]. In addition, working memory [48], short-term memory [49] and divided attention [35] are markedly impaired in patients with childhood chronic fatigue syndrome, which may lead to school refusal and is characterized by reduced intrinsic academic motivation [49,50]. Therefore, in order to prevent intrinsic academic motivation from decreasing, early detection of the signs of decrease in intrinsic academic motivation is crucial. The present findings suggest that capacity for verbal memory is related to the prevalence of decrease in intrinsic academic motivation in junior high school students. Therefore, measurement of capacity for verbal memory is useful for objectively evaluating the extent of decrease in intrinsic academic motivation. In particular, the digit span forward test and *kana *pick-out test are appropriate for evaluation of capacity for verbal memory in this age group.

## Limitations

The present study has three limitations. First, the study was performed with a limited number of participants. To generalize our results, studies involving a large number of participants are required. Second, we are unable to draw conclusions regarding cause-and-effect relationships because of the cross-sectional nature of our findings. Third, we neither excluded developmental disabilities from analyses nor measured socioeconomic or psychological status or intelligence quotient.

## Conclusions

The present findings suggest that decreases in short-term memory or working memory capacity and story comprehension during divided attention processing are significantly correlated with the prevalence of decrease in intrinsic academic motivation among 7th-, 8th-, and 9th-grade students in junior high school. These findings suggest that capacity for verbal memory influences the severity of decrease in intrinsic academic motivation in junior high school students. Our next study will have a longitudinal cohort design enabling identification of risk or predictive factors, in particular cognitive functions, for decreased motivation in the transition period from elementary to junior high school.

## List of abbreviations

ANOVA: Analysis of variance; CI: Confidence interval; fMRI: functional Magnetic Resonance Imaging; mATMT: modified Advanced Trail Making Test; OR: Odds ratio; RT: Reaction time; SD: Standard deviation; y.o.: years of age.

## Competing interests

The authors declare that they have no competing interests.

## Authors' contributions

KM took part in planning and designing the experiment and cognitive tests, corrected the data, performed the data analyses and drafted the manuscript. MT contributed to the design and planning of experiment and cognitive tests, corrected the data, and helped performing the data analyses and drafting the manuscript. SF contributed to the design and planning of experiment and corrected the data. KIM contributed to the design planning of experiment and recruited the participants. YW took part in the planning and designing the experiment and cognitive tests and helped drafting the manuscript. All authors read and approved the final manuscript.
